# Effects of Structural and Energetic Disorders on Charge Transports in Crystal and Amorphous Organic Layers

**DOI:** 10.1038/s41598-018-23204-w

**Published:** 2018-03-26

**Authors:** Furitsu Suzuki, Shosei Kubo, Tatsuya Fukushima, Hironori Kaji

**Affiliations:** Institute for Chemical Research, Kyoto University, Uji, Kyoto, 611-0011 Japan

## Abstract

Understanding charge transports in organic films is important for both fundamental science and practical applications. Here, contributions of off-diagonal (structural) and diagonal (energetic) disorders to charge transports were clarified using molecular-based multiscale simulation. These disorders, important for understanding charge transport in organic systems, are investigated by comparing crystal and amorphous aggregates of *N*,*N*′-diphenyl-*N*,*N*′-bis(1-naphthyl)-1,1′-biphenyl-4,4′-diamine (NPD). Although NPD has been used as a hole transport material, it also exhibits comparable electron mobility experimentally. The experimental mobility and its electric field dependence in amorphous layers were reasonably reproduced by the multiscale simulation, confirming the electron transport properties of NPD. We assumed that the structural disorder would lower mobilities; however, the mobilities were found to be independent of the degree of structural disorder. Energetic disorder markedly lowered charge mobility instead. Charge migration in crystals was dominated by maximum electronic coupling pairs, whereas small electronic coupling pairs significantly contributed to charge transport in amorphous aggregate.

## Introduction

Understanding the charge transport properties of organic thin films is of great interest not only for fundamental science but also for practical applications in organic electronics such as organic light-emitting diodes (OLEDs), organic solar cells, and organic thin-film transistors^1^. Materials with high charge mobility have attracted much attention for use in these devices because they can greatly contribute to device performance. Organic molecules in devices are often in amorphous states, and charge transport in such systems is considered to occur by intermolecular charge hopping. Some models have been proposed to explain the charge transport in organic amorphous aggregates^2–4^. For example, the Gaussian disorder model^4^, one of the most well-known models for charge transport, describes the macroscopic charge transport process in amorphous organic solids. However, this model lacks a microscopic (molecular-level) description of charge transport processes. Recently, charge transport simulations based on Marcus theory^5^ have been conducted for amorphous aggregates^6–17^, to reproduce experimental charge mobilities. However, the microscopic behaviour of charge transport in organic amorphous aggregates is still not fully understood.

Organic amorphous aggregates possess structural distributions by nature, such as distribution of intermolecular distance and that of relative orientation between adjacent molecules. The inherent structural distribution in an organic aggregate is usually called off-diagonal disorder or structural disorder. However, the effect of structural disorder on charge-hopping phenomena has not been fully considered at the molecular level. In this study, we perform charge transport simulations of crystalline and amorphous structures of *N*,*N*′-diphenyl-*N*,*N*′-bis(1-naphthyl)-1,1′-biphenyl-4,4′-diamine (NPD), a widely used hole transport material in OLEDs. A crystal and amorphous aggregate of NPD are used to exemplify ordered and disordered structures with negligible and large structural disorder, respectively. Energy levels of hopping sites are also distributed in amorphous aggregates; this distribution in energy is usually termed diagonal disorder or energetic disorder. We investigate the effect of energetic disorder as well as structural disorder on charge transfer through multiscale simulation by explicitly taking into account the organic molecule. Another purpose of this study is to examine the origin of bipolar charge transport in NPD. Although NPD is generally considered as a hole transport material^18–26^, it has been found to exhibit unexpectedly high electron mobility^27–31^ in addition to its favourable hole transporting ability.

## Results

**Figure 1:**
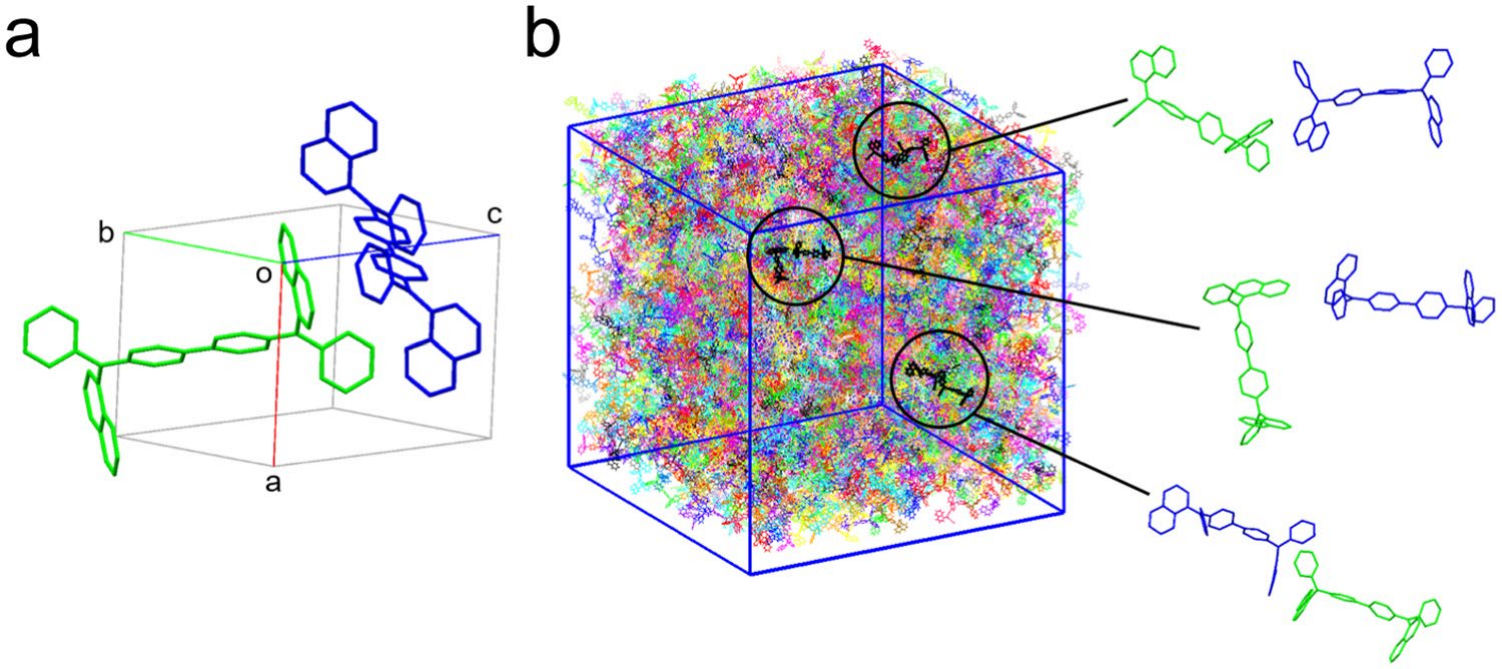
Structures of crystalline and amorphous NPD. (**a**) Crystal structure of NPD (cry-NPD). The molecules labelled I and II are crystallographically independent. (**b**) MD-constructed amorphous structure consisting of 4000 NPD molecules (amo-NPD). Examples of intermolecular packing in amo-NPD are also shown.

For the NPD crystal, the structure determined by X-ray diffraction reported by Cheng *et al*.^32^ was used without further structure optimisation (denoted as “cry-NPD”, Fig. 1a). An amorphous aggregate of NPD was constructed by molecular dynamics (MD) simulation of a system composed of 4000 NPD molecules in a cubic cell (denoted as “amo-NPD”, Fig. 1b). Figure 1b also exhibits three kinds of relative orientations in molecular pairs in amo-NPD as examples, showing various intermolecular packings. Supplementary Fig. S1 compares torsion angle distributions in amo-NPD and cry-NPD. They are significantly different due to the different nature of amorphous and crystalline aggregates. The reorganisation energies were calculated for molecules in the aggregated structure (*λ*_agg_^+^ and *λ*_agg_^−^ for hole and electron transport, respectively) by the quantum mechanics/molecular mechanics (QM/MM) method^33,34^. The electronic couplings for hole and electron transfer (*H*_*ij*_^+^ and *H*_*ij*_^−^, respectively) were calculated according to ref.^35^. The computational details were also provided in Methods section. Using the values of *H*_*ij*_^+^, *H*_*ij*_^−^, *λ*_agg_^+^ and *λ*_agg_^−^ obtained above, the rate constants for hole and electron transfer (*k*_*ij*_^+^ and *k*_*ij*_^−^, respectively) were calculated according to the following equation based on Marcus theory^5^,

1 $${k}_{ij}=\frac{4{\pi }^{2}}{h}{H}_{ij}^{2}\frac{1}{\sqrt{4\pi \lambda {k}_{{\rm{B}}}T}}\exp [-\frac{(\lambda +({\rm{\Delta }}{E}_{j}-{\rm{\Delta }}{E}_{i})-qF{\rm{\Delta }}{x}_{ij}{)}^{2}}{4\lambda {k}_{{\rm{B}}}T}]$$

where *T* is the temperature, *h* is Planck’s constant, *k*_B_ is Boltzmann’s constant, *q* is the charge of the carrier, *F* is the applied external electric field strength and Δ*x*_*ij*_ is the distance between molecules *i* and *j* along the direction of the applied electric field. Δ*E*_*i*_ and Δ*E*_*j*_ are the energies for the relevant two molecules *i* and *j*, respectively. On the basis of the method by Uratani *et al*.^17^, these energies were calculated with taking into account the difference in Coulombic interaction between neighbouring molecules.

**Figure 2:**
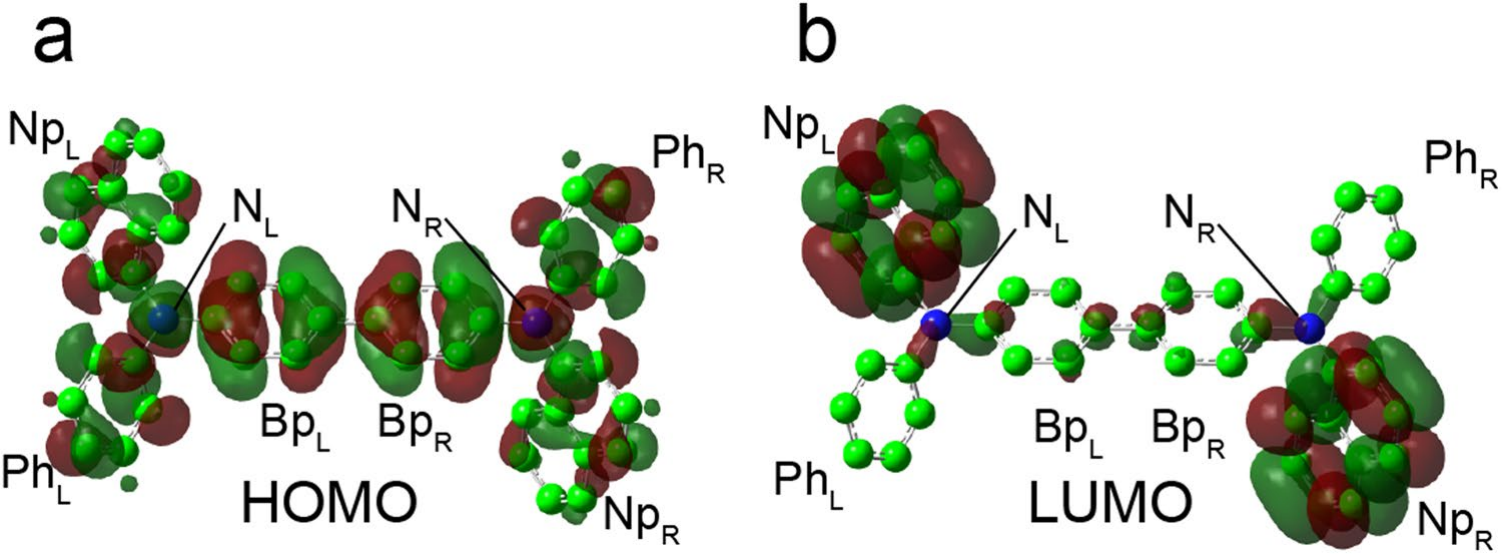
HOMO and LUMO of an NPD molecule in cry-NPD. Ph, Np and Bp denote the phenyl, naphthyl and biphenyl segments, respectively. N denotes nitrogen atoms. The subscripts L and R denote left and right, respectively. HOMO and LUMO of the molecule used as the initial structure to construct amo-NPD are shown in Supplementary Fig. S2.

**Figure 3:**
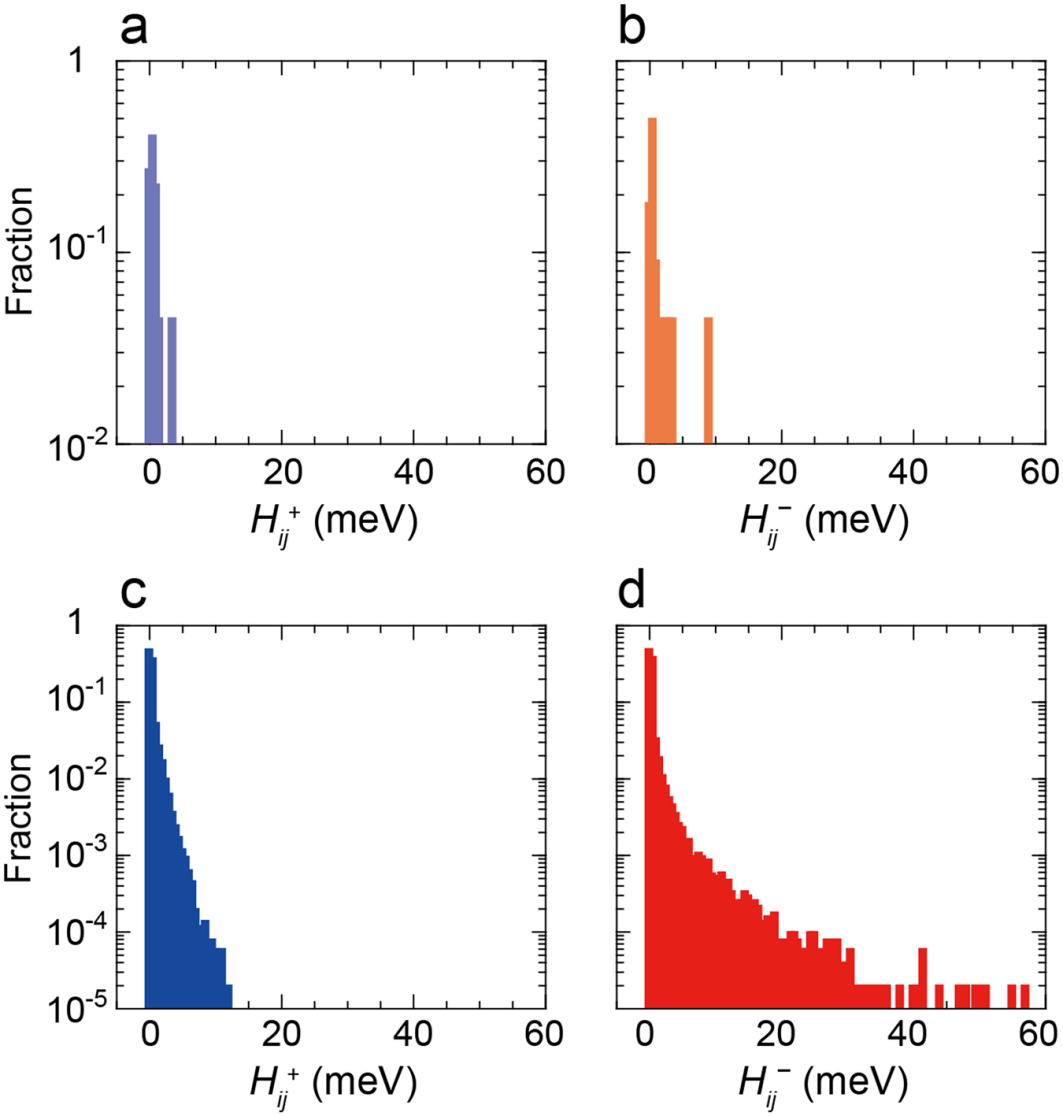
Distribution of *H*_*ij*_ values (structural disorder). *H*_*ij*_ for a (**a**) hole in cry-NPD, (**b**) electron in cry-NPD, (**c**) hole in amo-NPD and (**d**) electron in amo-NPD. Maximum *H*_*ij*_ values are 3.1, 8.8, 11.9 and 57.0 meV for (**a**), (**b**), (**c**) and (**d**), respectively.

Figure 2 shows the highest occupied molecular orbital (HOMO) and lowest unoccupied molecular orbital (LUMO) of molecule I in cry-NPD (Fig. 1a). The HOMO and LUMO of molecule II were similar. Compared with the HOMO, which is relatively delocalised over the entire molecule, the LUMO is mainly localised on two outer naphthyl segments with only a small contribution along the central part, nitrogen atoms and biphenylene groups, as shown in Supplementary Table S1. A DFT-optimised NPD molecule used as the initial structure to construct amo-NPD provided a similar result (Supplementary Fig. S2 and Supplementary Table S1). The calculated *H*_*ij*_ values for cry-NPD and amo-NPD are presented in Fig. 3. The maximum *H*_*ij*_ values for electron (*H*_*ij*_^−^_max_ = 8.8 meV for cry-NPD and 57.0 meV for amo-NPD) are larger than those for hole (*H*_*ij*_^+^_max_ = 3.1 meV for cry-NPD and 11.9 meV for amo-NPD), suggesting that NPD possesses good electron transport properties. The calculated value of *λ*_agg_ for electron transport is smaller than that of hole transport (Supplementary Table S2), which also indicates good electron transport properties. Taking a closer look at the case of cry-NPD reveals that the pair with the *H*_*ij*_^−^_max_ of 8.8 meV has close face-to-face intermolecular contact between naphthyl segments (Supplementary Fig. S3a). In contrast, the molecular pair with *H*_*ij*_^+^_max_ shown in Supplementary Fig. S3b has smaller intermolecular overlaps between orbitals (*H*_*ij*_^+^_max_ = 3.1 meV). The *H*_*ij*_ values of amo-NPD have a much larger distribution than those of cry-NPD (Fig. 3), and *H*_*ij*_^−^_max_ and *H*_*ij*_^+^_max_ of amo-NPD (57.0 and 11.9 meV, respectively; the molecular pairs are shown in Supplementary Fig. S4) are larger than those of cry-NPD. In the Gaussian disorder model^4^, *H*_*ij*_ is solely determined by intermolecular distance. However, no clear correlation was found between *H*_*ij*_ and intermolecular distance both for cry-NPD and for amo-NPD (Supplementary Fig. S5). This result demonstrates that intermolecular orientational packing and frontier orbital distribution as well as intermolecular distance have significant influences on *H*_*ij*_.

**Figure 4:**
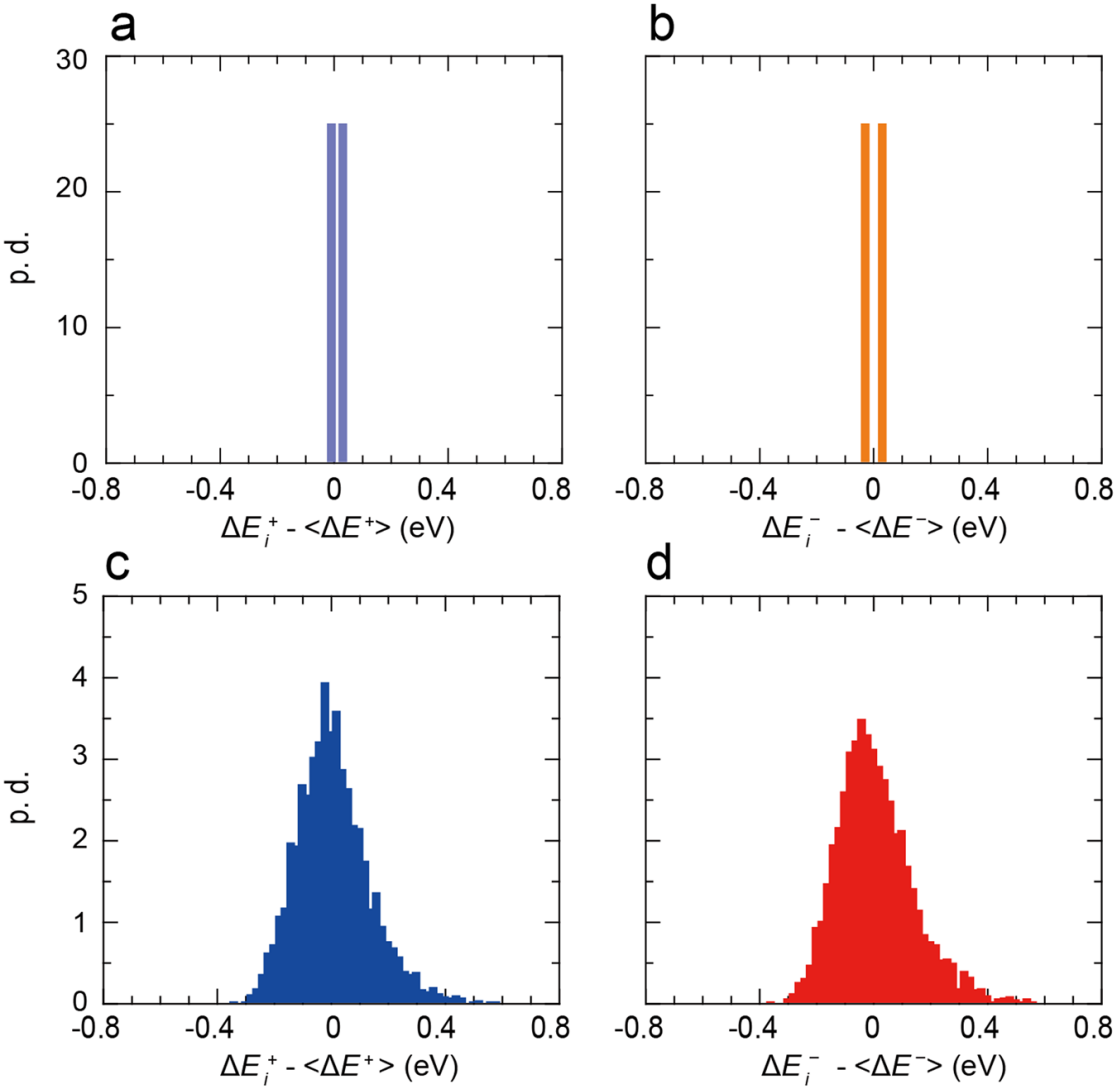
Distributions of site energies Δ*E*_*i*_ (energetic disorder). Probability densities for a (**a**) hole in cry-NPD, (**b**) electron in cry-NPD, (**c**) hole in amo-NPD and (**d**) electron in amo-NPD. $\langle {\rm{\Delta }}{E}^{+}\rangle$ and $\langle {\rm{\Delta }}{E}^{-}\rangle$ are average values of Δ*E*_*i*_^+^ and Δ*E*_*i*_^−^, respectively.

Figure 4 shows the energetic disorders in cry-NPD and amo-NPD. The two crystallographically different molecules in the unit cell of cry-NPD, molecule I and II in Fig. 1a, have slightly different site energies from each other; the differences were 0.059 and 0.036 eV for electron and hole transports, respectively. Compared with that of cry-NPD, the energetic disorder in amo-NPD is much larger (Fig. 4c and d), reflecting varied intermolecular interactions of surrounding molecules in the amorphous aggregate. In the Gaussian disorder model, the energetic disorder is assumed to be a Gaussian. However, in our work, no assumption was made for the distribution function. Figure 4c,d reveal that the distribution obtained in our calculation was slightly skewed to the higher energy region, but mostly Gaussian. The effects of structural disorder (distribution of *H*_*ij*_) and energetic disorder (distribution of Δ*E*_*i*_) on the charge transport in NPD will be shown below.

**Figure 5:**
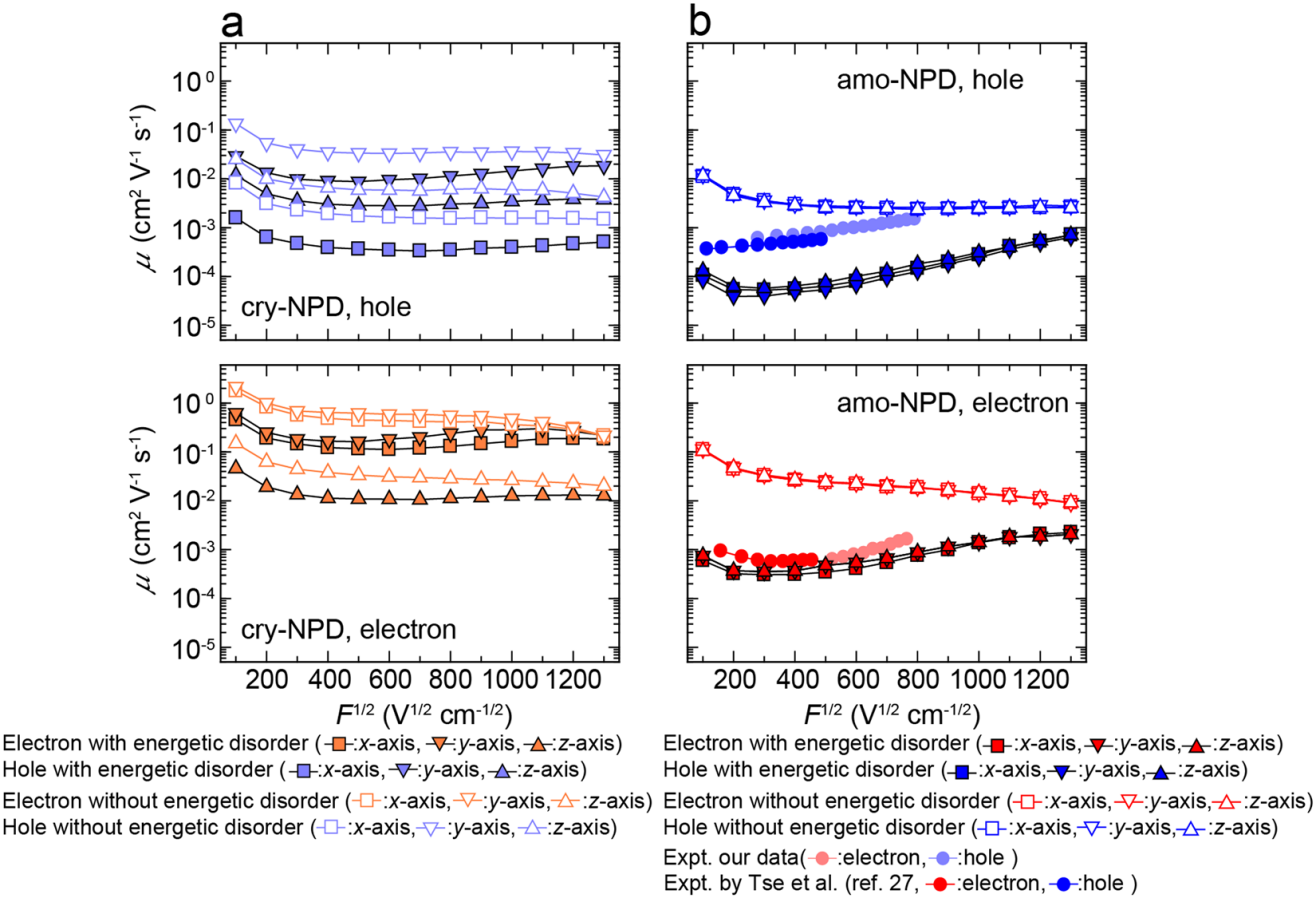
Calculated charge mobilities *μ*. Calculated *μ* for (**a**) cry-NPD and (**b**) amo-NPD. Filled symbols: calculated with energetic disorder. Open symbols: calculated without energetic disorder. Experimental data (filled circles) are also shown.

On the basis of *k*_*ij*_ calculated by equation (1), we performed charge transport simulations by kinetic Monte Carlo method for both cry-NPD and amo-NPD. Figure 5a displays the dependence of the calculated hole (upper figure) and electron (lower figure) mobilities on the square root of electric field ($\sqrt{F}$) for cry-NPD with and without considering energetic disorder. The charge mobilities along the *x*-, *y*- and *z*-axes (Supplementary Fig. S6 for respective axes) are different irrespective of the inclusion of energetic disorder, showing that charge transport is anisotropic in cry-NPD. The charge mobilities differ by one to two orders of magnitude depending on the direction of the applied electric field. Electron mobility is higher than hole mobility by one to two orders of magnitude, reflecting the larger *H*_*ij*_ and smaller *λ* for electron transport than those for hole transport (see above). Reflecting the small energetic disorder in cry-NPD (Fig. 4a,b), slight decrease of charge mobility was found when energetic disorder is considered (Fig. 5a). Figure 5b shows the dependence of the hole and electron mobilities on $\sqrt{F}$ for amo-NPD. The difference in charge mobility along each axis is negligible for amo-NPD, reflecting the isotropic nature of the amorphous NPD system. Experimental charge mobilities are also presented in Fig. 5b. Our experimental data and those reported by Tse *et al*.^27^ agree well with each other. For hole transport, positive $\sqrt{F}$ dependence, so-called Poole–Frenkel dependence, is experimentally observed. In contrast, electron mobility shows interesting $\sqrt{F}$ dependence; the mobility first decreases and then increases with increasing $\sqrt{F}$. Compared with the experimental results, the charge mobility calculated without energetic disorder is one to two orders of magnitude higher at low $\sqrt{F}$ and has negative $\sqrt{F}$ dependence. When we consider energetic disorder, the hole mobility was one order of magnitude smaller than that obtained experimentally. In contrast, experimental electron mobilities are well reproduced by the multiscale simulation when the energetic disorder is considered. It should be emphasised that the experimentally observed $\sqrt{F}$ dependence was reproduced well for electron transport, including the negative $\sqrt{F}$ dependence at $\sqrt{F}$ lower than 300 V^1/2^ cm^−1/2^. From equation (1), *F* appears in the exponential term as $\lambda +({\rm{\Delta }}{E}_{j}-{\rm{\Delta }}{E}_{i})-qF{\rm{\Delta }}{x}_{ij}$. At low electric field, $qF{\rm{\Delta }}{x}_{ij}$ is relatively small compared to $\lambda +({\rm{\Delta }}{E}_{j}-{\rm{\Delta }}{E}_{i})$. In this case, *k*_*ij*_ and therefore the velocity of electrons (*v*) do not strongly depend on *F*. As a result, charge mobility (which equals *v*/*F*) decreases with increasing *F*.

Comparison of Fig. 5a and b revealed that the calculated charge mobilities without considering energetic disorder and their $\sqrt{F}$ dependence in amo-NPD are comparable to those in cry-NPD (within the direction-dependent charge mobilities of cry-NPD). This holds for both hole and electron transfer, indicating that the structural disorder of NPD does not play a critical role in its charge mobility and applied electric field dependence. The result, “*structural disorder in amorphous systems does not decrease charge mobility*” is contrary to our expectation. Upon introduction of energetic disorder, a slight (one order of magnitude or smaller) decrease of charge mobility was found in the crystals, and the $\sqrt{F}$ dependence was mostly preserved. In contrast, for the amorphous NPD, the charge mobilities dramatically decreased when energetic disorder was considered, especially at low electric field, resulting in a positive $\sqrt{F}$ dependence, so-called Poole–Frenkel behaviour. These results show that it is not the structural disorder but the energetic disorder that causes the Poole–Frenkel behaviour of amorphous NPD.

**Figure 6:**
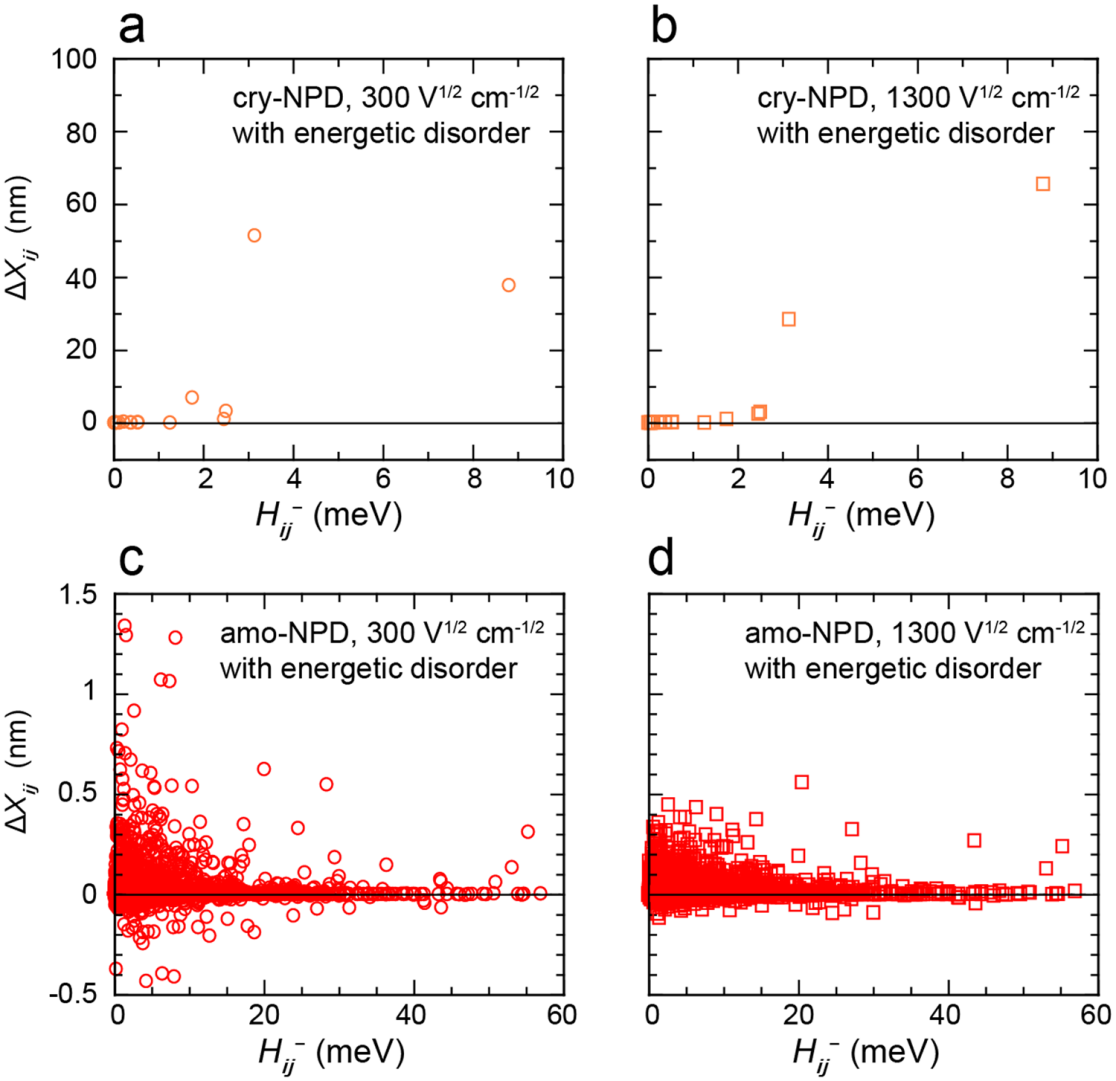
Correlation between Δ*X*_*ij*_ and *H*_*ij*_^−^. Energetic disorder was considered in all calculations. At $\sqrt{F}$ of (**a**) 300 V^1/2^ cm^−1/2^ in cry-NPD, (**b**) 1300 V^1/2^ cm^−1/2^ in cry-NPD, (**c**) 300 V^1/2^ cm^−1/2^ in amo-NPD and (**d**) 1300 V^1/2^ cm^−1/2^ in amo-NPD.

Hereafter, the molecular-level analysis will be carried out for electron transport because it reproduced the experimental results well. The same is essentially true for hole transport, although the simulated mobilities were about one order of magnitude smaller than experimental ones. Here, we introduce the migration distance for respective pairs, Δ*X*_*ij*_, as follows:

2 $${\rm{\Delta }}{X}_{ij}={N}_{{\rm{d}}{\rm{i}}{\rm{f}}{\rm{f}}}\cdot {\rm{\Delta }}{x}_{ij},$$

where Δ*x*_*ij*_ is the distance between neighbouring relevant molecules *i* and *j* along the electric field. As shown later, charges hop in backward as well as forward directions. *N*_diff_ is the number of difference of forward and backward charge hops between the molecules *i* and *j*. In this study, we calculated Δ*X*_*ij*_ for the average of 10,000 trials of charge transport. Figure 6a,b show the correlation between Δ*X*_*ij*_ and *H*_*ij*_^−^ for cry-NPD with energetic disorder (the correlation for the case in which energetic disorder was ignored is summarised in Supplementary Fig. S7). For cry-NPD, large Δ*X*_*ij*_ were obtained for the molecular pairs with maximum and second maximum *H*_*ij*_^−^. Figure 6c,d show the correlation between Δ*X*_*ij*_ and *H*_*ij*_^−^ for amo-NPD with energetic disorder. In this case, molecular pairs with small *H*_*ij*_^−^ contribute markedly to charge transport (which provide large Δ*X*_*ij*_), especially at $\sqrt{F}$ = 300 V^1/2^ cm^−1/2^, although negative values of Δ*X*_*ij*_ are also found. The negative Δ*X*_*ij*_ means that the molecular pair conveys charge in opposite direction to the electric field vector. The contribution of molecular pairs with small *H*_*ij*_^−^ decreased at high electric field (Fig. 6d). Using Δ*X*_*ij*_, we can estimate why charge mobility in amorphous aggregates is comparable to that in crystalline aggregates when energetic disorder is not included. Figure 3 clearly shows that the *H*_*ij*_^+^ and *H*_*ij*_^−^ are widely distributed in amo-NPD. The distributions of *H*_*ij*_^+^ and *H*_*ij*_^−^ in cry-NPD are narrower and the maximum values are far smaller compared to the case of amo-NPD. In addition, Supplementary Fig. S7c,d indicate that not only small *H*_*ij*_ pairs but also large *H*_*ij*_ pairs effectively convey charges in amo-NPD. To summarise, *H*_*ij*_ values in amorphous aggregates are widely distributed due to the structural disorder, but almost the all distributed *H*_*ij*_ pairs contribute the charge transport. Moreover, most of the *H*_*ij*_ values in amo-NPD are larger than those in cry-NPD. This would be the reason that amorphous aggregates have good charge mobility comparable to crystalline systems under the condition that energetic disorder is not considered.

**Figure 7:**
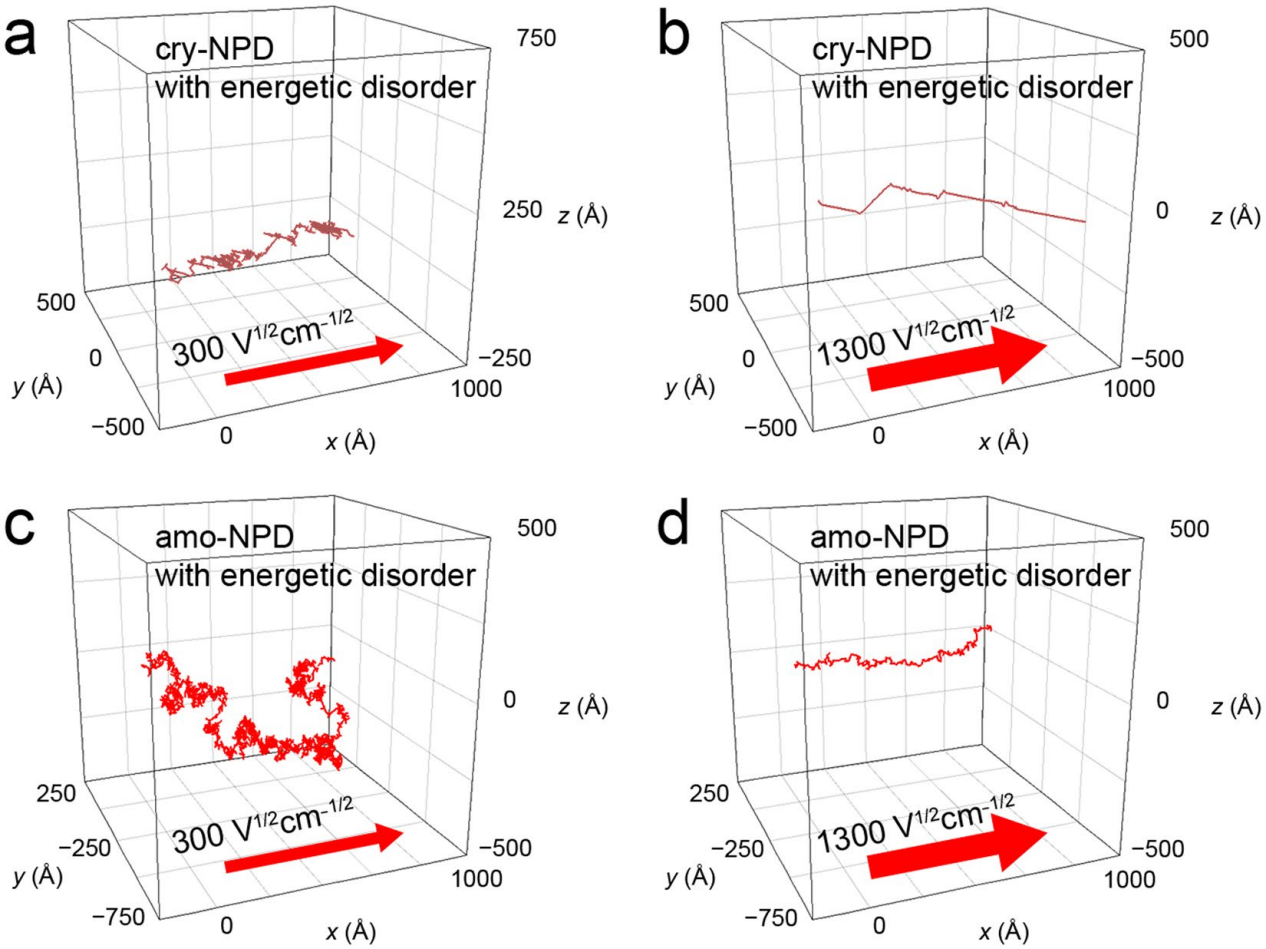
Electron transport trajectories considering energetic disorder. At $\sqrt{F}$ of (**a**) 300 V^1/2^ cm^−1/2^ in cry-NPD, (**b**) 1300 V^1/2^ cm^−1/2^ in cry-NPD, (**c**) 300 V^1/2^ cm^−1/2^ in amo-NPD and (**d**) 1300 V^1/2^ cm^−1/2^ in amo-NPD. The electric field (indicated by red arrows) was applied in the direction of the *x*-axis.

To understand the differences in charge hopping for cry-NPD and amo-NPD, we carried out further molecular-level analysis. Table 1 shows the number of charge hops in the forward direction (*N*_fwd_) and backward direction (*N*_bwd_), along with their sum (*N*_all_ = *N*_fwd_ + *N*_bwd_) and difference (*N*_diff_ = *N*_fwd_ − *N*_bwd_) during electron transport with energetic disorder. The analysis for electron transport without energetic disorder is summarised in Supplementary Table S3. These values are for the pairs with the three highest Δ*X*_*ij*_ values. *N*_all_/*N*_diff_ in this table indicates the efficiency of charge hops of each pair. In the present kinetic Monte Carlo simulations, charges travel between the two electrodes with a separation distance of 100 nm. Therefore, the sum of Δ*X*_*ij*_ for all the pairs is 100 nm. In the case of cry-NPD with energetic disorder, the pairs with the two highest *H*_*ij*_ mostly contribute to charge hopping at 300 V^1/2^ cm^−1/2^ (the sum of Δ*X*_*ij*_ is 51.4 + 37.7 = 89.1 nm). For all the pairs, both forward (*N*_fwd_) and backward (*N*_bwd_) charge hoppings are found at 300 V^1/2^ cm^−1/2^; the values of *N*_all_/*N*_diff_ are 4.5–5.7, indicating that charge hops forward once every 4–6 hops. In contrast, at 1300 V^1/2^ cm^−1/2^, the *N*_all_/*N*_diff_ values for the pairs with three highest Δ*X*_*ij*_ values are almost 1, indicating that charges hop only in the forward direction at 1300 V^1/2^ cm^−1/2^. Table 1 also shows the case of amo-NPD. In amo-NPD, many molecular pairs are used during the charge transport and thus Δ*X*_*ij*_ values for respective molecular pairs in amo-NPD are much smaller than those of cry-NPD. In amo-NPD, Δ*X*_*ij*_ is relatively small for the pair with maximum electronic coupling (*H*_*ij*_^−^_max_ = 57.0 meV) regardless of the inclusion of energetic disorder (smaller than 0.094 nm, Supplementary Figs S8a–S11a). The largest Δ*X*_*ij*_ value (Δ*X*_*ij*,max_) is determined by the pairs that have a smaller *H*_*ij*_ compared to *H*_*ij*_^−^_max_ (Supplementary Figs S8b–S11b). When the energetic disorder is considered, Δ*X*_*ij*,max_ at 300 V^1/2^ cm^−1/2^ is provided by the pair with *H*_*ij*_ = 1.42 meV (Table 1 and Supplementary Fig. S8b). The second and third largest Δ*X*_*ij*_ are provided by the pairs with *H*_*ij*_ of 1.59 and 8.19 meV, respectively (Table 1 and Supplementary Fig. S8c). At 300 V^1/2^ cm^−1/2^, *N*_fwd_ and *N*_bwd_ are almost same order of magnitude for both cry-NPD and amo-NPD (Table 1). This result indicates that charge hopping not only in the forward and but also in the backward direction is common feature for both crystal and amorphous systems at low electric field. Finally, Fig. 7 compares electron transport trajectories in cry-NPD and amo-NPD considering energetic disorder (similar trajectories were found for hole transport). For the case of cry-NPD at 300 V^1/2^ cm^−1/2^ (Fig. 7a), an electron reaches the counter electrode through mostly linear routes, with some small fluctuations; both forward and backward hops occur, as shown in Table 1. At 1300 V^1/2^ cm^−1/2^ (Fig. 7b), an almost linear route is followed because of the repeated use of the molecular pairs with large *H*_*ij*_, although the route is tilted from the electric field direction (*x*-axis). In this case, backward hops seldom occurred, as mentioned above. In contrast, a much more complicated route is observed in amo-NPD at 300 V^1/2^ cm^−1/2^ (Fig. 7c). Forward and backward charge hoppings occur in various directions, indicating diffusive-like behaviour in amo-NPD. At a higher applied electric field of 1300 V^1/2^ cm^−1/2^, the charge in amo-NPD reaches the counter electrode using more linear routes than that at 300 V^1/2^ cm^−1/2^ (Fig. 7d).

**Table 1:** *N*_fwd_, *N*_bwd_, *N*_all_ and *N*_diff_ for the molecular pairs with the three largest Δ*X*_*ij*_ values. These values were calculated for electron transfer considering energetic disorder (averaged for 10,000 charges).

	$\sqrt{{\boldsymbol{F}}}$ (V^1/2^ cm^−1/2^)	*H*_*ij*_^−^ (meV)	Number of hops				*N*_all_/*N*_diff_	Δ*X*_*ij*_ (nm)
			*N*_fwd_	*N*_bwd_	*N*_all_	*N*_diff_		
cry-NPD	300	3.2	167.0	117.0	283.0	49.8	5.7	51.4
		8.8	123.0	86.1	209.0	36.6	5.7	37.7
		1.8	12.3	7.8	20.1	4.5	4.5	6.9
	1300	8.8	63.6	0.1	63.7	63.5	1.0	65.5
		3.2	27.6	0.0	27.6	27.5	1.0	28.4
		2.5	6.1	0.6	6.7	5.6	1.2	3.0
amo-NPD	300	1.4	2.5	1.5	4.0	0.9	4.3	1.34
		1.6	3.5	2.8	6.3	0.7	8.9	1.29
		8.2	559.0	558.0	1116.0	1.1	948.0	1.28
	1300	20.5	1.2	0.9	2.1	0.3	6.4	0.56
		2.6	0.4	0.0	0.4	0.3	1.1	0.45
		6.3	0.6	0.2	0.8	0.4	2.1	0.43

In conclusion, we performed molecular-based multiscale charge transport simulations to investigate the effects of disorders in amorphous systems, in particular, the influence of structural disorder on charge transport in NPD, by comparing crystalline and amorphous aggregates. NPD has been used as a hole-transport material in OLEDs, but was found to exhibit electron as well as hole transport ability. Experimentally-obtained charge mobility and its field dependence in the amorphous thin layer, including interesting feature of the negative applied field dependence at low electric field, were quantitatively reproduced for electron transport by our multiscale simulation. The existence of “*energetic*” disorder markedly lowered charge mobility for amorphous NPD, especially at low electric field. However, the comparison of the crystalline and amorphous systems unexpectedly revealed that “*structural*” disorder does not obviously decrease charge mobility. Analysis of the actual contribution of each molecular pair to the migration distance revealed a substantial difference between crystalline and amorphous systems. Charge migration was basically dominated by the molecular pairs with maximum and second maximum electronic coupling in the crystalline aggregate, whereas the molecular pairs with relatively small electronic coupling contributed to the charge transport in the amorphous aggregate. These findings on the effects of structural and energetic disorders on charge-hopping phenomena at the molecular level should aid the design of materials with superior charge transport properties.

## Methods

### Computational details

The MD simulations of amorphous systems were performed using the LAMMPS program^36^. For the MD calculation, the Dreiding force field^37^ was used and the bond lengths of the DFT-optimised molecular structure were applied as the equilibrium bond length parameters. The DFT calculation (B3LYP functional^38^ and 6–31G(d) basis set) was performed on the Gaussian 09 program package^39^. To mimic the deposition process, the initial MD simulation was performed in the constant-volume, constant-temperature (NVT) ensemble at a density of 0.1 g cm^−3^ and temperature of 573 K for 10 ps. Then, the MD simulation was performed under the constant-pressure, constant-temperature (NPT) ensemble for 1.0 ns at 298 K until the density of the system became constant. Finally, the structure was geometry optimised to eliminate the structure deviation originating from vibrational motion during MD calculations. The final density of the amorphous structure was 0.95 g cm^−3^ (the density of cry-NPD was 1.22 g cm^−3^). The amorphous structure thus obtained, amo-NPD, is shown in Fig. 1b.

*λ*_agg_^+^ and *λ*_agg_^−^ were calculated by QM/MM method^33,34^, which includes the effect of intermolecular interactions^17,40^. In the QM region, DFT with B3LYP functional and 6–31G(d) basis set was used and the Dreiding force field were used in the MM region for surrounding molecules within a distance of 30 Å. For comparison, the reorganisation energies for an isolated NPD molecule (*λ*_iso_) were also calculated according to previous reports^16,17,41,42^. The values of *λ*_agg_ and *λ*_iso_ are summarised in Supplementary Table S2.

*H*_*ij*_^+^ and *H*_*ij*_^−^ were calculated by the following equation.

3 $${H}_{ij}=\frac{{\beta }_{ij}-({\alpha }_{i}+{\alpha }_{j}){S}_{ij}/2}{1-({S}_{ij}{)}^{2}},$$

where ${\alpha }_{i}=\langle {\psi }_{i}|{\hat{H}}_{ij}|{\psi }_{i}\rangle$, ${\alpha }_{j}=\langle {\psi }_{j}|{\hat{H}}_{ij}|{\psi }_{j}\rangle$, ${\beta }_{ij}=\langle {\psi }_{i}|{\hat{H}}_{ij}|{\psi }_{j}\rangle$, and ${S}_{ij}=\langle {\psi }_{i}|{\psi }_{j}\rangle$. Here, ${\hat{H}}_{ij}$ is the electronic Hamiltonian of a dimer system composed of molecules *i* and *j*. ${\psi }_{i}$ and ${\psi }_{j}$ are the HOMO or LUMO of the isolated molecules *i* and *j*, respectively. Molecular pairs with a centre-to-centre distance within 25 Å are considered in the *H*_*ij*_ calculation.

The charge transport simulations for both cry-NPD and amo-NPD were performed based on kinetic Monte Carlo calculations. The distance (*L*) of charge migration along the applied electric field ***F*** is 100 nm and 10,000 trials of charge transports were simulated to calculate the travelling time (*t*). The mobility *μ* was calculated from *μ* = *L*/(*tF*), where *F* = |***F***|.

### Data availability

The datasets generated during and/or analysed during the current study are available from the corresponding author on reasonable request.

## Electronic supplementary material

Supplementary Information
