# Supplementary material for: Effects of Structural and Energetic Disorders on Charge Transports in Crystal and Amorphous Organic Layers
Source: Sci Rep. 2018 Mar 26;8:5203. doi: 10.1038/s41598-018-23204-w (PMC5979998; doi:10.1038/s41598-018-23204-w)
Supplement: Supplementary file 1 — Supplementary Information [file 41598_2018_23204_MOESM1_ESM.docx]

Supplementary Information for:
Effects of Structural and Energetic Disorders on Charge Transports in Crystal and Amorphous Organic Layers

Furitsu Suzuki, Shosei Kubo, Tatsuya Fukushima, Hironori Kaji*

Institute for Chemical Research, Kyoto University, Uji, Kyoto 611-0011, Japan

* kaji@scl.kyoto-u.ac.jp


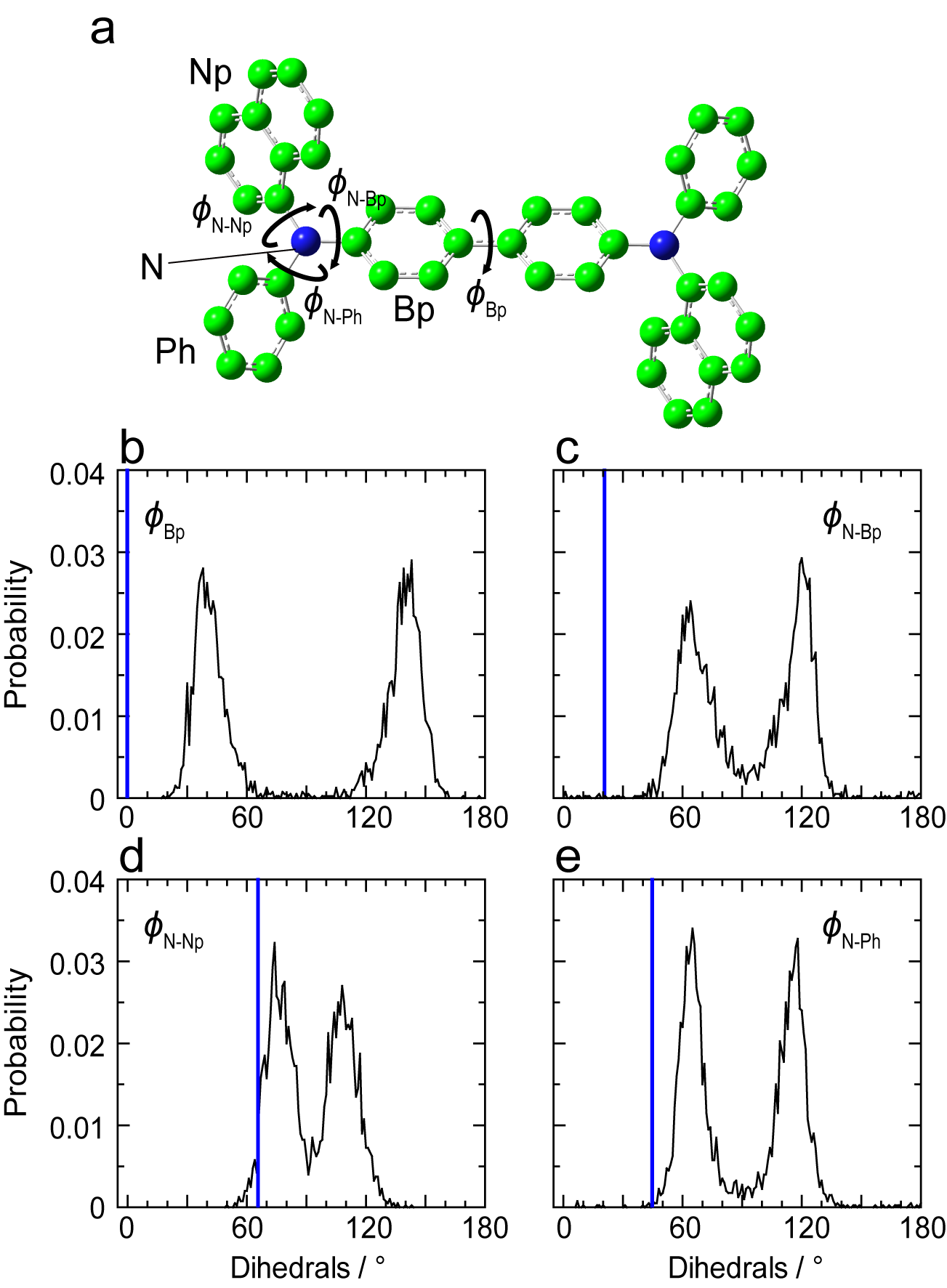


Supplementary Figure S1 | Torsion angle distributions in amo-NPD. (a) Molecular structure of NPD, showing the assignments of respective torsion angles. Torsion angle distributions of (b) $\phi_{\mathrm{Bp}}$, (c) $\phi_{N-Bp}$, (d) $\phi_{N-Np}$, and (e) $\phi_{N-Ph}$. Torsion angles for cry-NPD were also shown as blue vertical lines (The torsion angles provided by two crystallography independent molecules are superposed).

Supplementary Table S1 | Percentage contributions of respective moieties to the HOMO and LUMO. The contributions of the nitrogen, biphenylene, phenyl and naphthyl moieties are denoted as N, Bp, Ph and Np, respectively. The subscript L or R indicates the left or right side, respectively, of the molecule in Fig. 2 and Supplementary Fig. S2. Upper: molecule I in cry-NPD. Lower: DFT-optimised NPD used as the initial structure to construct amo-NPD.

|  |  | N_L_ | N_R_ | Bp_L_ | Bp_R_ | Ph_L_ | Ph_R_ | Np_L_ | Np_R_ |
| --- | --- | --- | --- | --- | --- | --- | --- | --- | --- |
| I | HOMO | 17.6 | 17.7 | 18.5 | 18.4 | 7.5 | 7.5 | 6.4 | 6.4 |
|  | LUMO | 0.2 | 0.2 | 1.5 | 1.5 | 0.5 | 0.5 | 47.8 | 48.0 |
| DFT-optimised | HOMO | 16.8 | 16.3 | 17.0 | 16.8 | 8.5 | 8.4 | 8.1 | 8.2 |
|  | LUMO | 0.2 | 0.2 | 2.2 | 2.2 | 0.3 | 0.4 | 45.7 | 48.8 |


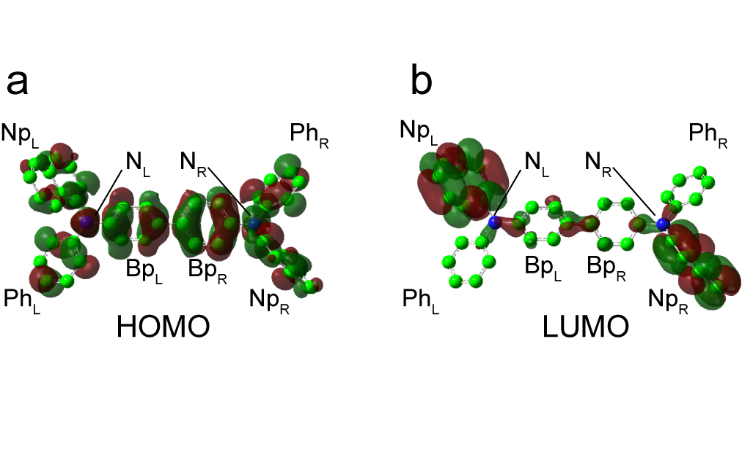


Supplementary Figure S2 | HOMO and LUMO of DFT-optimised NPD used as the initial structure to construct amo-NPD. Ph, Np and Bp denote the phenyl, naphthyl and biphenyl segments, respectively. N denotes nitrogen atoms. The subscripts L and R denote left and right, respectively.

Supplementary Table S2 | Reorganisation energies. Calculated for an isolated molecule (*λ*_iso_) and for a molecule including the intermolecular packing effect in cry-NPD or amo-NPD (*λ*_agg_). All values are in eV.

|  | cry-NPD | | amo-NPD | |
| --- | --- | --- | --- | --- |
|  | *λ*_iso_ | *λ*_agg_ | *λ*_iso_ | *λ*_agg_ |
| Hole | 0.323 | 0.200 | 0.286 | 0.289 |
| Electron | 0.163 | 0.130 | 0.164 | 0.151 |


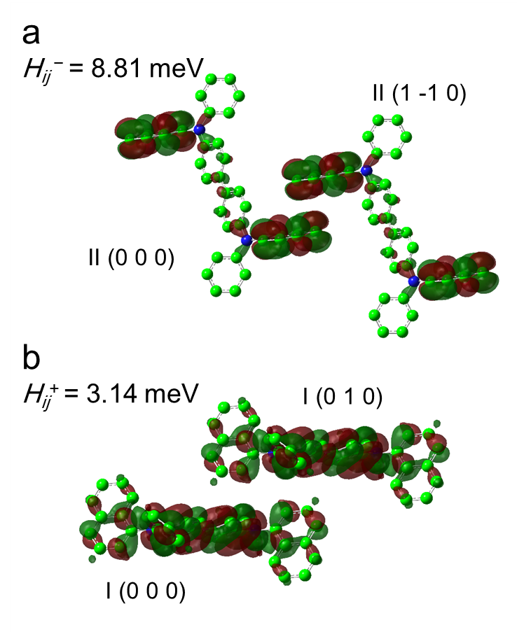


Supplementary Figure S3 | Molecular pairs with the largest *H_ij_* values in cry-NPD. For (a) electron and (b) hole transport.


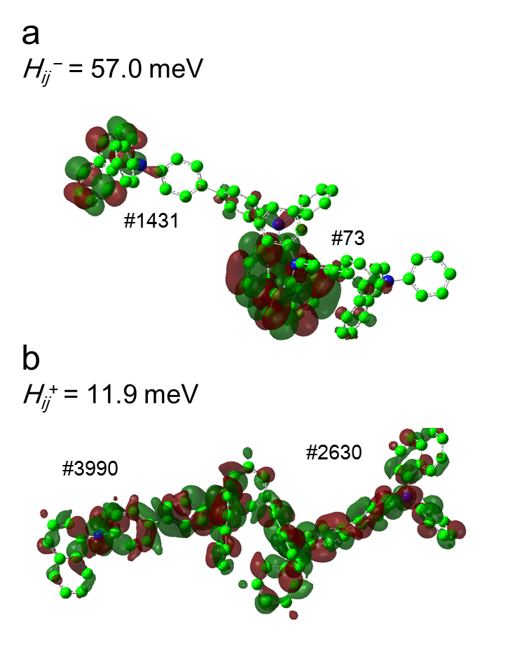


Supplementary Figure S4 | Molecular pairs with the largest *H_ij_* values in amo-NPD. For (a) electron and (b) hole transport.


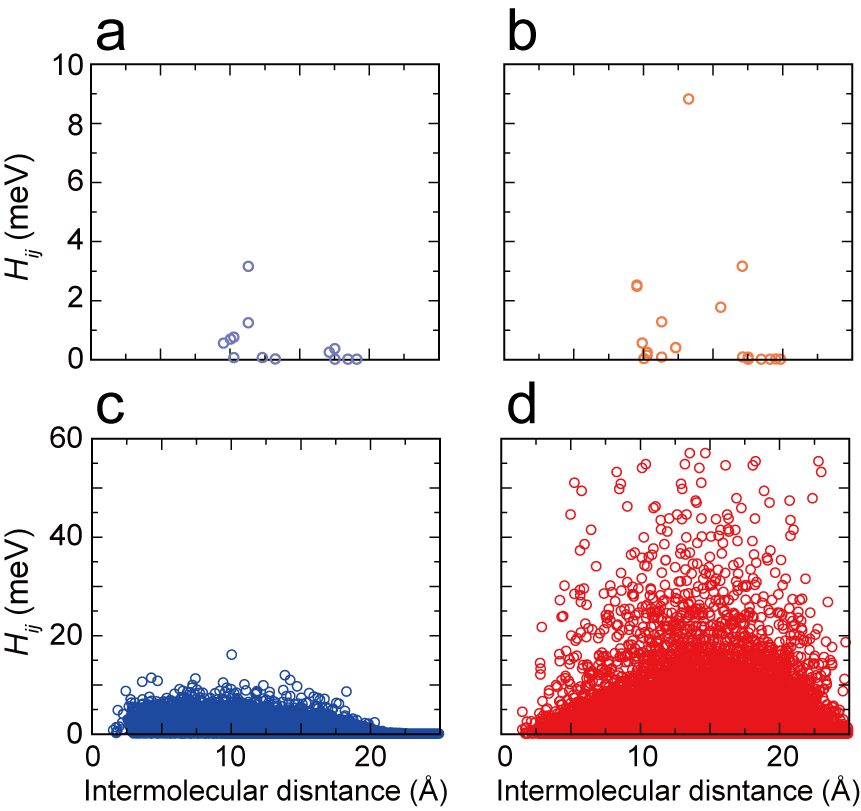


Supplementary Figure S5 | Correlation between *H_ij_* and intermolecular distance. For (a) hole transfer in cry-NPD, (b) electron transfer in cry-NPD, (c) hole transfer in amo-NPD and (d) electron transfer in amo-NPD.


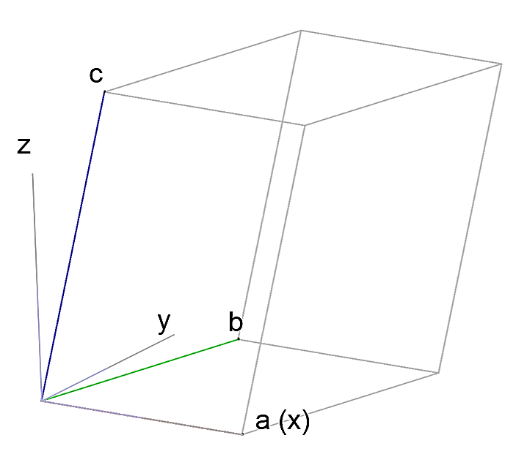


Supplementary Figure S6 | Unit cell axes (*a*, *b* and *c*) and orthogonal coordinate axes (*x*, *y* and *z*) for cry-NPD. The crystal system is triclinic with unit cell parameters of *a* = 10.3077 Å, *b* = 11.3545 Å, *c* = 14.4778 Å, *α* = 82.339°, *β* = 77.657° and *γ* = 75.526°. Note that the *a*-axis is in the same direction as the *x*-axis.


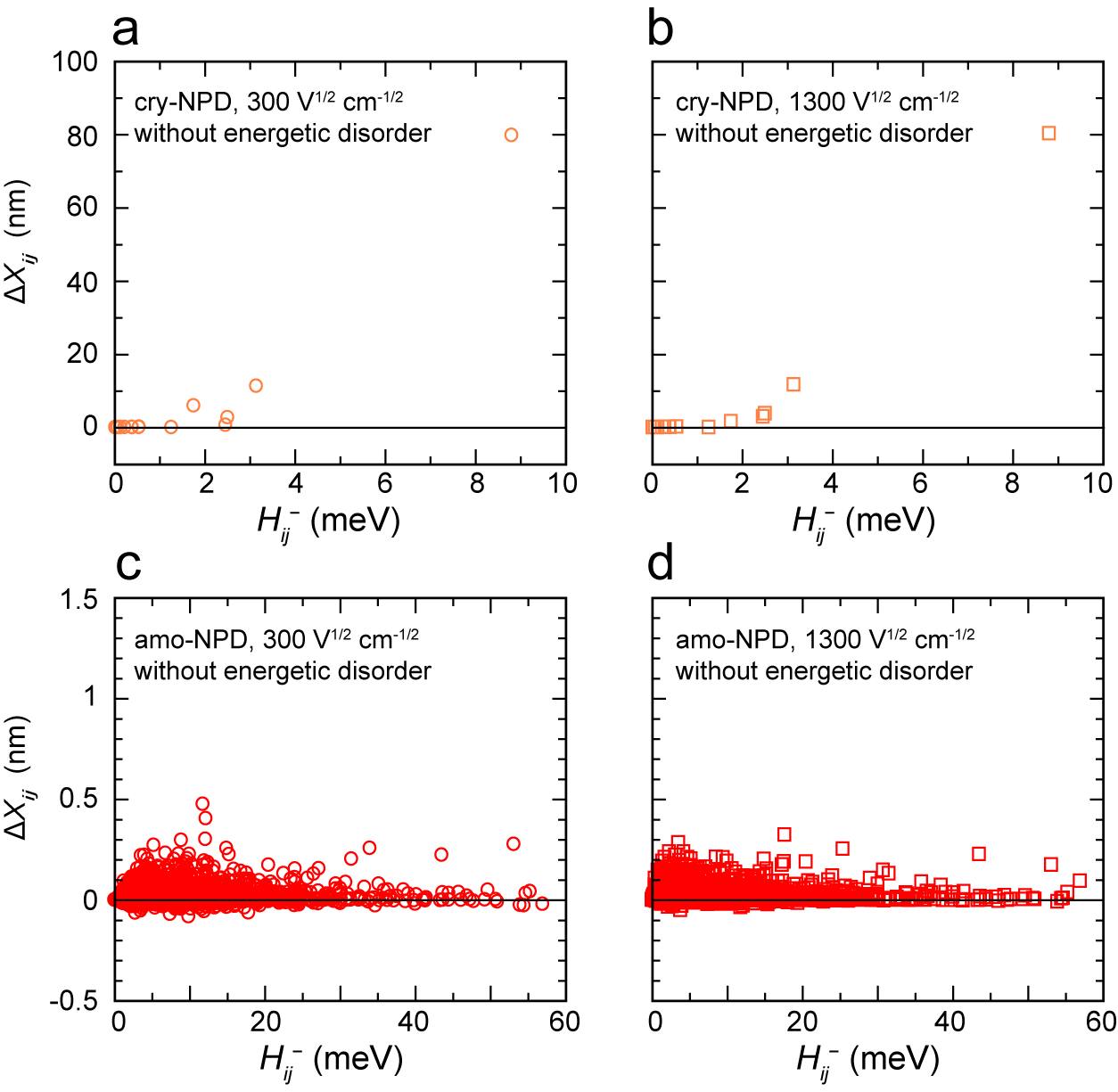


Supplementary Figure S7 | Correlation between Δ*X_ij_* and *H_ij_*^−^. Energetic disorder was ignored in all these calculations. At $\sqrt{F}$ of (a) 300 V^1/2^ cm^−1/2^ in cry-NPD, (b) 1300 V^1/2^ cm^−1/2^ in cry-NPD, (c) 300 V^1/2^ cm^−1/2^ in amo-NPD and (d) 1300 V^1/2^ cm^−1/2^ in amo-NPD.

Supplementary Table S3 | *N*_fwd_, *N*_bwd_, *N*_all_ and *N*_diff_ for the molecular pairs with the three largest Δ*X_ij_* values for electron transport.

|  | Energetic  disorder | $\sqrt{F}$  (V^1/2^ cm^−1/2^) | *H_ij_*^−^  (meV) | Number of hops | | | | *N*_all_ / *N*_diff_ | Δ*X_ij_*  (nm) |
| --- | --- | --- | --- | --- | --- | --- | --- | --- | --- |
|  |  |  |  | *N*_fwd_ | *N*_bwd_ | *N*_all_ | *N*_diff_ |  |  |
| cry-  NPD | On | 300 | 3.2 | 167.0 | 117.0 | 283.0 | 49.8 | 5.7 | 51.4 |
|  |  |  | 8.8 | 123.0 | 86.1 | 209.0 | 36.6 | 5.7 | 37.7 |
|  |  |  | 1.8 | 12.3 | 7.8 | 20.1 | 4.5 | 4.5 | 6.9 |
|  |  | 1300 | 8.8 | 63.6 | 0.1 | 63.7 | 63.5 | 1.0 | 65.5 |
|  |  |  | 3.2 | 27.6 | 0.0 | 27.6 | 27.5 | 1.0 | 28.4 |
|  |  |  | 2.5 | 6.1 | 0.6 | 6.7 | 5.6 | 1.2 | 3.0 |
|  | Off | 300 | 8.8 | 257.0 | 179.0 | 436.0 | 77.4 | 5.6 | 79.8 |
|  |  |  | 3.2 | 36.3 | 25.3 | 61.5 | 11.0 | 5.6 | 11.3 |
|  |  |  | 1.8 | 11.1 | 7.2 | 18.3 | 3.9 | 4.7 | 6.0 |
|  |  | 1300 | 8.8 | 77.9 | 0.1 | 78.0 | 77.8 | 1.0 | 80.2 |
|  |  |  | 3.2 | 11.3 | 0.0 | 11.4 | 11.3 | 1.0 | 11.7 |
|  |  |  | 2.5 | 7.3 | 0.2 | 7.6 | 7.1 | 1.1 | 3.8 |
| amo-  NPD | On | 300 | 1.4 | 2.5 | 1.5 | 4.0 | 0.9 | 4.3 | 1.34 |
|  |  |  | 1.6 | 3.5 | 2.8 | 6.3 | 0.7 | 8.9 | 1.29 |
|  |  |  | 8.2 | 559.0 | 558.0 | 1116.0 | 1.1 | 948.0 | 1.28 |
|  |  | 1300 | 20.5 | 1.2 | 0.9 | 2.1 | 0.3 | 6.4 | 0.56 |
|  |  |  | 2.6 | 0.4 | 0.0 | 0.4 | 0.3 | 1.1 | 0.45 |
|  |  |  | 6.3 | 0.6 | 0.2 | 0.8 | 0.4 | 2.1 | 0.43 |
|  | Off | 300 | 11.8 | 2.9 | 2.6 | 5.5 | 0.4 | 15.8 | 0.48 |
|  |  |  | 12.2 | 4.3 | 4.0 | 8.4 | 0.3 | 26.5 | 0.40 |
|  |  |  | 12.1 | 2.5 | 2.3 | 4.7 | 0.2 | 22.8 | 0.30 |
|  |  | 1300 | 17.7 | 0.4 | 0.1 | 0.5 | 0.3 | 1.6 | 0.32 |
|  |  |  | 3.5 | 0.2 | 0.0 | 0.2 | 0.2 | 1.0 | 0.28 |
|  |  |  | 25.4 | 0.2 | 0.0 | 0.2 | 0.2 | 1.1 | 0.25 |


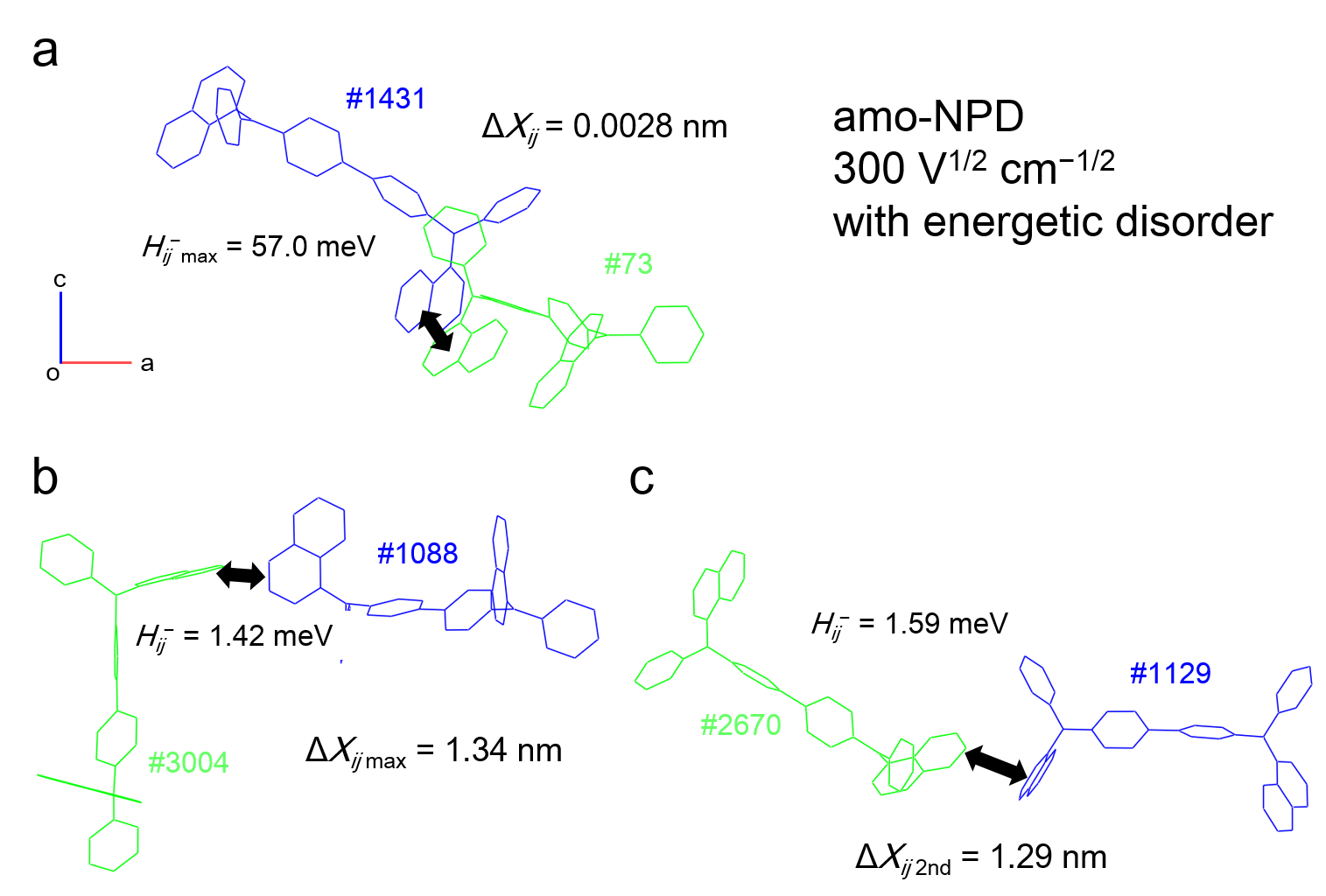


Supplementary Figure S8 | *H_ij_*^−^ and Δ*X_ij_* for selected molecular pairs in amo-NPD with energetic disorder and at $\sqrt{\boldsymbol{F}}$ = 300 V^1/2^ cm^−1/2^. Molecular pairs with (a) largest *H_ij_*^−^, (b) largest Δ*X_ij_* and (c) second largest Δ*X_ij_* for electron transfer along the *a*-axis (in the same direction as the *x*-axis).


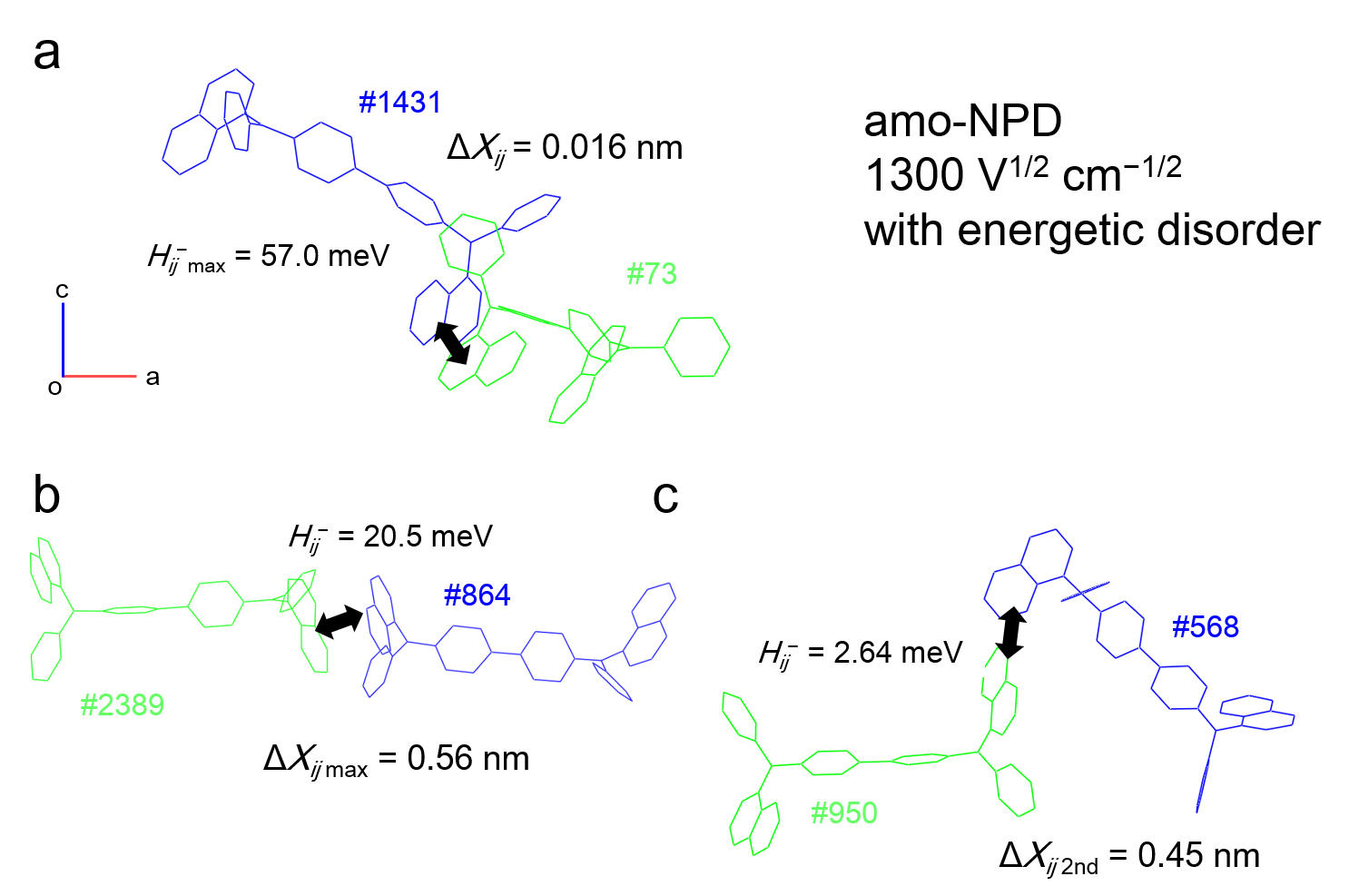


Supplementary Figure S9 | *H_ij_*^−^ and Δ*X_ij_* for selected molecular pairs in amo-NPD with energetic disorder and at $\sqrt{\boldsymbol{F}}$ = 1300 V^1/2^ cm^−1/2^. Molecular pairs with (a) largest *H_ij_*^−^, (b) largest Δ*X_ij_* and (c) second largest Δ*X_ij_* for electron transfer along the *a*-axis (in the same direction as the *x*-axis).


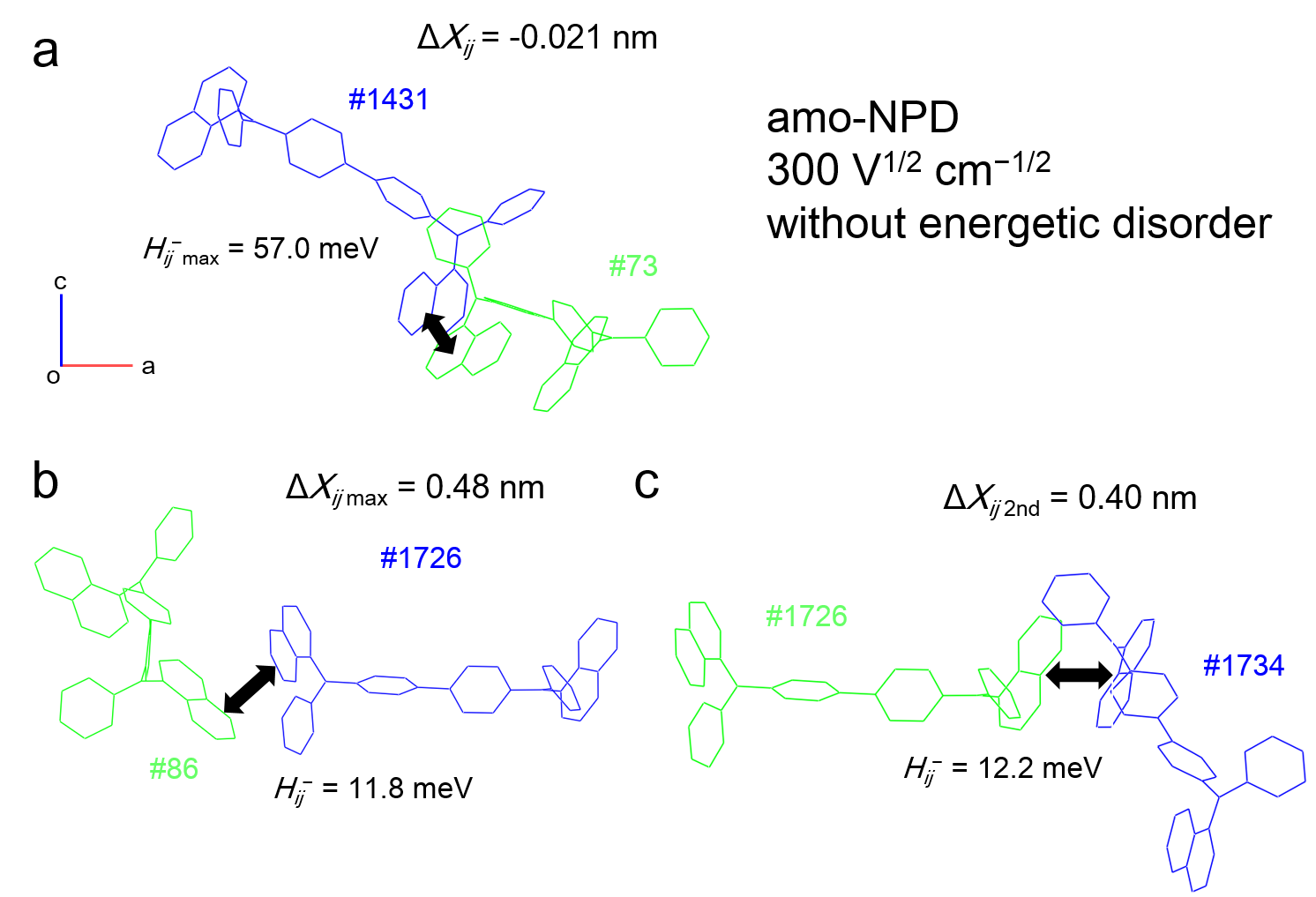


Supplementary Figure S10 | *H_ij_*^−^ and Δ*X_ij_* for selected molecular pairs in amo-NPD without energetic disorder and at $\sqrt{\boldsymbol{F}}$ = 300 V^1/2^ cm^−1/2^. Molecular pairs with (a) largest *H_ij_*^−^, (b) largest Δ*X_ij_* and (c) second largest Δ*X_ij_* for electron transfer along the *a*-axis (in the same direction as the *x*-axis).


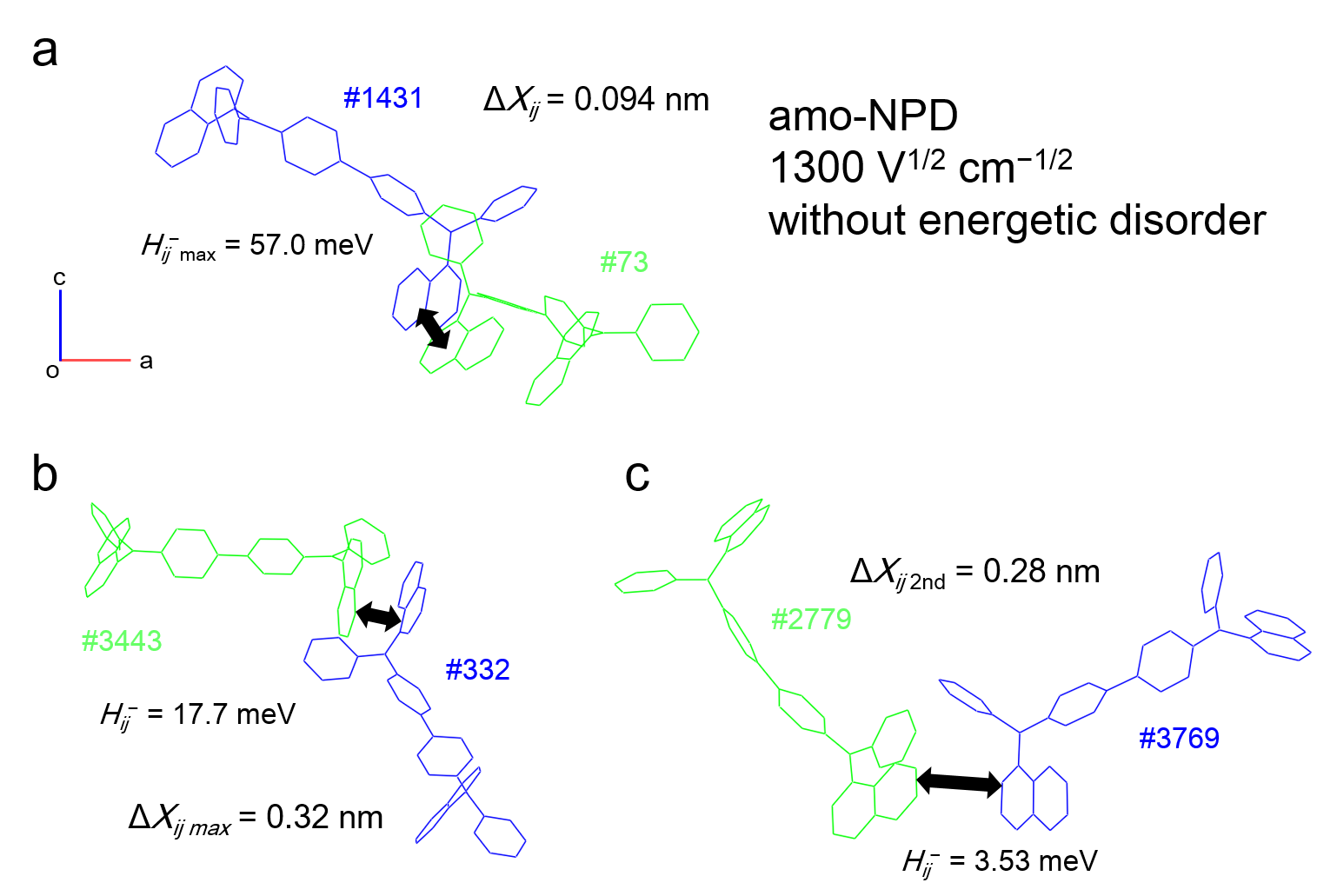


Supplementary Figure S11 | *H_ij_*^−^ and Δ*X_ij_* for selected molecular pairs in amo-NPD without energetic disorder and at $\sqrt{\boldsymbol{F}}$ = 1300 V^1/2^ cm^−1/2^. Molecular pairs with (a) largest *H_ij_*^−^, (b) largest Δ*X_ij_* and (c) second largest Δ*X_ij_* for electron transfer along the *a*-axis (in the same direction as the *x*-axis).


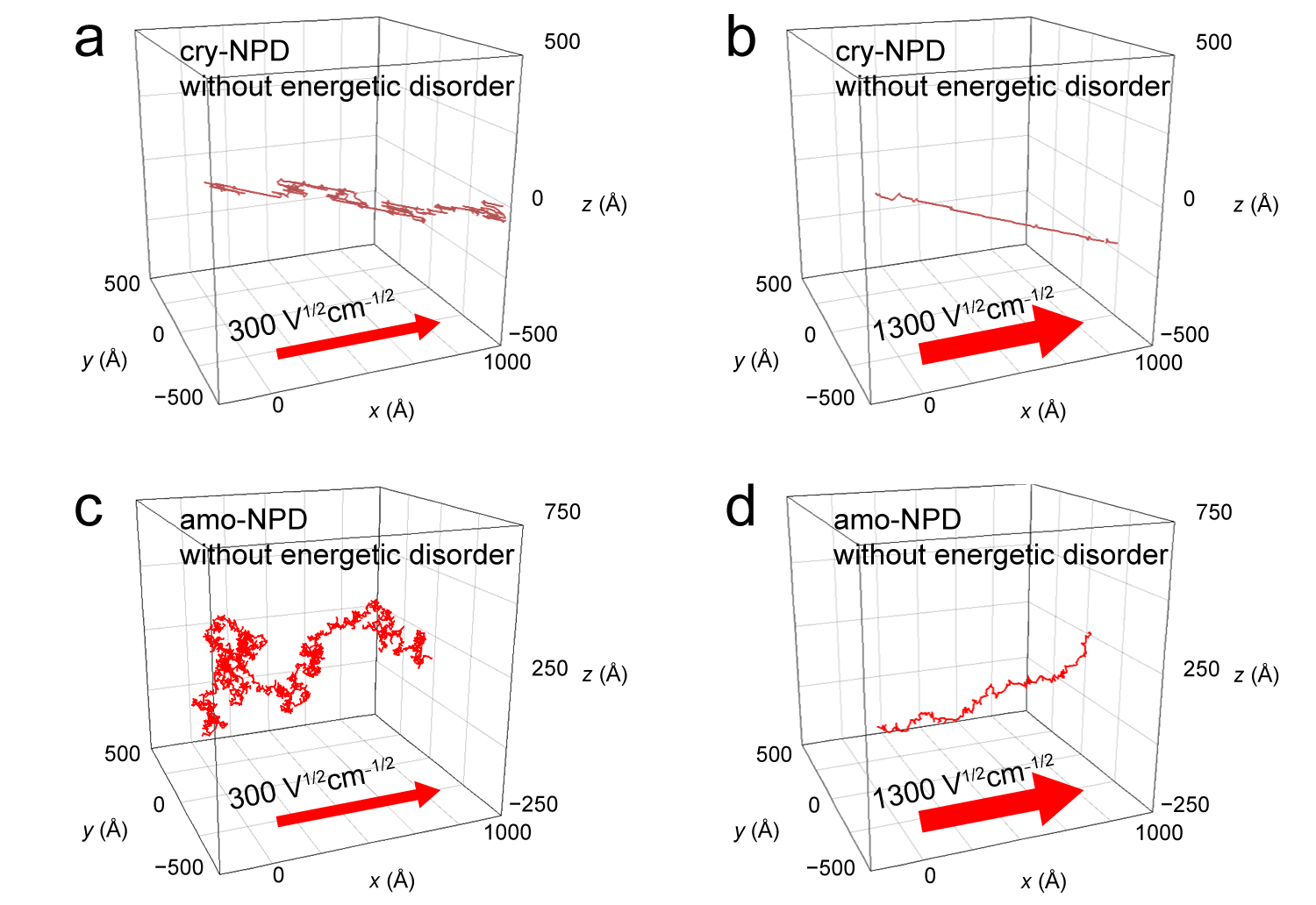


Supplementary Figure S12 | Electron transport trajectories without considering energetic disorder. At $\sqrt{F}$ of (a) 300 V^1/2^ cm^−1/2^ in cry-NPD, (b) 1300 V^1/2^ cm^−1/2^ in cry-NPD, (c) 300 V^1/2^ cm^−1/2^ in amo-NPD and (d) 1300 V^1/2^ cm^−1/2^ in amo-NPD. The electric field was applied in the direction of the *x*-axis.

# Experimental Methods

## Mobility measurements

Hole and electron mobilities for vacuum-deposited sublimed NPD were measured at 300 K with time-of-flight (TOF) equipment (TOF-401-3, Sumitomo Heavy Industries Advanced Machinery Co., Ltd., Japan). The device structure for TOF samples was indium tin oxide (ITO; 50 nm) / NPD (5.1 μm) / Al (20 nm), which was fabricated with deposition apparatus with a multi-chamber system (SE-4260, ALS Technology, Japan). After Al deposition, the TOF sample was encapsulated with a glass cap incorporated with a calcium oxide sheet using epoxy glue in a N_2_-filled glove box. The photocarriers in the NPD film were generated by a N_2_ gas laser (KEC-150, Ushio Optical Systems Co., Ltd., Japan) with a wavelength of 337 nm.
